# Contribution of Satellite-Derived Aerosol Optical Depth PM_2.5_ Bayesian Concentration Surfaces to Respiratory-Cardiovascular Chronic Disease Hospitalizations in Baltimore, Maryland

**DOI:** 10.3390/atmos11020209

**Published:** 2020-02-18

**Authors:** John T. Braggio, Eric S. Hall, Stephanie A. Weber, Amy K. Huff

**Affiliations:** 1 Mt. Diablo Analytical Solutions and Reporting Institute, Walnut Creek, CA 94595, USA; 2 U.S. Environmental Protection Agency (EPA), Research Triangle Park, NC 27709, USA; 3 Battelle Memorial Institute, Columbus, OH 43201, USA; 4 I. M. Systems Group, 5825 University Research Ct, Suite 3250, College Park, MD 20740, USA

**Keywords:** PM_2.5_, AOD-PM_2.5_, CMAQ, case-crossover, IP hospitalizations, ED visits: asthma, myocardial infarction, heart failure, season

## Abstract

The fine particulate matter baseline (PMB), which includes PM_2.5_ monitor readings *fused* with Community Multiscale Air Quality (CMAQ) model predictions, using the Hierarchical Bayesian Model (HBM), is less accurate in rural areas without monitors. To address this issue, an upgraded HBM was used to form four experimental aerosol optical depth (AOD)-PM_2.5_ concentration surfaces. A case-crossover design and conditional logistic regression evaluated the contribution of the AOD-PM_2.5_ surfaces and PMB to four respiratory-cardiovascular hospital events in all 99 12 km^2^ CMAQ grids, and in grids with and without ambient air monitors. For all four health outcomes, only two AOD-PM_2.5_ surfaces, one not kriged (PMC) and the other kriged (PMCK), had significantly higher Odds Ratios (ORs) on lag days 0, 1, and 01 than PMB in all grids, and in grids without monitors. In grids with monitors, emergency department (ED) asthma PMCK on lag days 0, 1 and 01 and inpatient (IP) heart failure (HF) PMCK ORs on lag days 01 were significantly higher than PMB ORs. Warm season ORs were significantly higher than cold season ORs. Independent confirmation of these results should include AOD-PM_2.5_ concentration surfaces with greater temporal-spatial resolution, now easily available from geostationary satellites, such as GOES-16 and GOES-17.

## 1. Introduction

The adverse effects of PM_2.5_ on the respiratory-cardiovascular system have been repeatedly confirmed [1–12]. Epidemiologic studies that assess exposure risk to different ambient PM_2.5_ concentration levels have relied on the U.S. Environmental Protection Agency’s (EPA) network of ground-based ambient air pollutant monitors [4,13–16]. Ambient air monitors are not equally spatially distributed, and most make measurements every 3 or 6 days [13,16–18]. There are also characterization errors in available PM_2.5_ concentration measurements due to limited instrument measurement precision and spatial heterogeneity [19].

In 2004, the U.S. Centers for Disease Control and Prevention (CDC) and EPA established and logistically supported the CDC Public Health Air Surveillance Evaluation (PHASE) project [18,20–22]. One important PHASE project outcome was the development of the first-generation HBM that statistically fused PM_2.5_ monitor concentration readings with CMAQ PM_2.5_ model predictions [23–25]. In urban areas, PMB gives more “weight” to PM_2.5_ monitor readings than CMAQ PM_2.5_ model predictions. In rural areas, CMAQ PM_2.5_ model predictions exert more influence than PM_2.5_ monitor readings on PMB, since there are fewer monitors or no monitors. Ambient air monitors are usually found in urban areas. In the last 15 years, PMB has turned out to be a more representative PM_2.5_ concentration surface, compared to the interpolation of PM_2.5_ monitor data, as a method to resolve spatial gaps between ambient air monitors [16,18,22]. CDC subsequently incorporated PMB into its Environmental Public Health Tracking (EPHT) network of state and New York City partners [16,18,22,26]. To date, PMB has been used by federal and state epidemiologists completing EPHT projects in different parts of the US [16,18,22,26].

Within this decade, the availability and use of satellite AOD data have become more routine [6,16,27–31]. Newer generation satellite instruments measure AOD with increased temporal accuracy and finer spatial resolution [27,32–37]. AOD is a unitless measure of the scattering and absorption of visible light by aerosols (particles) in the atmosphere [38–40]. AOD data are, by definition, actual physical measurements, an improvement over CMAQ PM_2.5_ model predictions. Once AOD unitless measurements have been calibrated with actual PM_2.5_ readings from on-the-ground ambient air monitors, it is then possible to utilize the derived AOD-PM_2.5_ concentration readings to estimate *actual* ambient PM_2.5_ concentration in areas where there are no on-the-ground air monitors. The relationship between AOD measurements and on-the-ground measurements of PM_2.5_ concentration readings has been confirmed in available publications [16,27–29,39–44].

By incorporating AOD-PM_2.5_ concentration values into the currently utilized PMB, we hypothesized there would be a further improvement in the fused AOD-PM_2.5_-PMB surface [16]. Our intention, in this preliminary work, was to test this hypothesis by using these four experimental AOD-PM_2.5_ and PMB fused concentration surfaces with linked health outcome data from Baltimore, Maryland, and New York City, New York, in a case-crossover epidemiologic study design data files analyzed by using conditional logistic regression [16]. From the beginning, our expectation was to complete the Baltimore and New York City epidemiologic studies at the same time [16]. Unexpected circumstances related to restricted access to the Maryland emergency department (ED) and inpatient hospitalization IP confidential hospital data files, delayed the completion of the Baltimore AOD-PM_2.5_ study component, and this delay made it possible for the Baltimore investigators to examine additional questions, such as completing a fine-grain evaluation of areas with and without ambient PM_2.5_ monitors. The New York City study, published in 2016, did not find differences between the four experimental AOD-PM_2.5_ concentration surfaces and PMB [16].

These were the objectives of the Baltimore study: firstly, replicate the New York City study by using all CMAQ 12 km^2^ grids, and completing further analyses in grids with and without PM_2.5_ ambient air monitors. Secondly, determine if the four experimental AOD-PM_2.5_ concentration surfaces differed from PMB in grids with and without monitors. Thirdly, evaluate warm season versus cold season differences.

## 2. Methods

### 2.1. Description of Study Participants

This study used the same experimental methods described in the New York City publication [16]. The same procedural steps used in the two study sites are summarized in this Methods section, with more detail included on differences unique to the Baltimore study.

**Figure 1.**
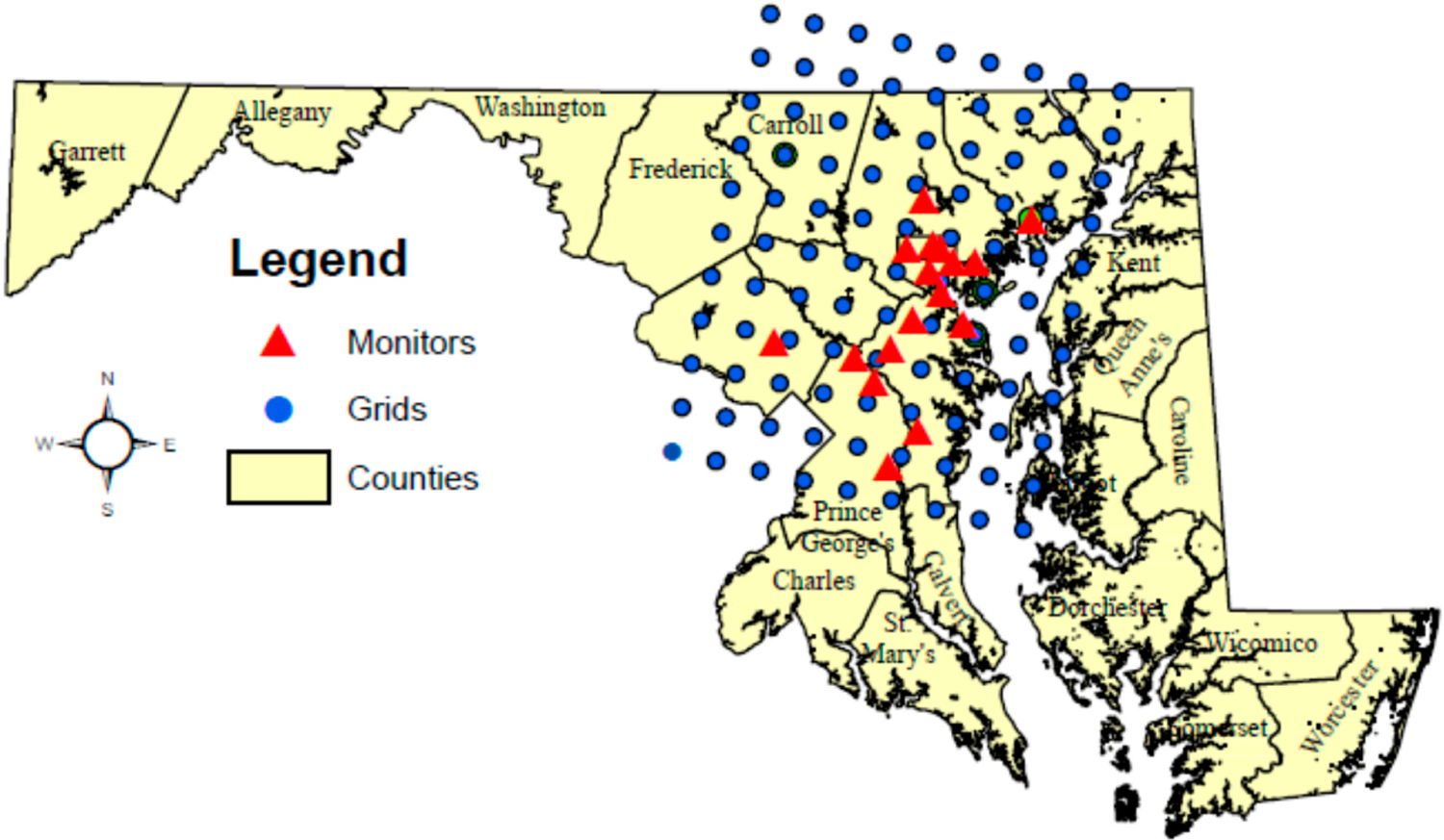
Map shows Maryland’s Counties and Baltimore City in the study area. The extent of the study area is defined by the 1–11 (south–north row) by 1–9 (west–east column) Community Multiscale Air Quality (CMAQ) 12 km^2^ grids (blue circles). The 17 Federal Reference Method (FRM) PM_2.5_ ambient air monitors are shown as red triangles. Baltimore City and Maryland Counties within the CMAQ grid boundaries provided the 2004–2006 respiratory-cardiovascular chronic disease hospital events included in this data analysis study.

Figure 1 is a map of the 11 (south–north) by 9 (west–east) 12 km^2^ CMAQ grid cells, shown in blue circles, and the l7 Federal Reference Method (FRM) PM_2.5_ ambient air monitors, shown in red triangles. Baltimore City had 6 PM_2.5_ ambient air monitors, more than in Anne Arundel (4), Prince George’s (3), Baltimore (2), Montgomery (1) and Harford (1) Counties. Other Counties in the study area did not have a single ambient air monitor. Table S1 (Supplementary Materials) includes more information on each of the 17 FRM PM_2.5_ ambient air monitors, e.g., CMAQ grid row-column identifier, site number, county name, city name, years in operation, between 2004–2006.

### 2.2. HBM Inputs

#### 2.2.1. Monitor PM_2.5_

Baltimore study area 2004–2006 24-hour average PM_2.5_ concentration data files were downloaded from the EPA Air Quality System (AQS) database [17]. The locations of the Maryland study area FRM PM_2.5_ monitors were mapped to the 12 km^2^ CMAQ grid system prior to their incorporation into the upgraded HBM. If a grid cell contained two or more monitor concentration readings on any given day, a single daily mean was computed and retained. Grid cells without monitor measurements on a given day were set to the “missing” data condition.

#### 2.2.2. CMAQ PM_2.5_

The 2004–2006 Baltimore study area CMAQ PM_2.5_ model predictions were obtained from EPA’s Community Modeling and Analysis System website [24,25]. CMAQ PM_2.5_ model predictions did not have missing values.

#### 2.2.3. AOD-PM_2.5_

Moderate Resolution Imaging Spectroradiometer (MODIS) Collection 5 AOD 2004–2006 data files, at 10 km^2^ resolution, were downloaded from the National Aeronautics and Space Administration (NASA) Level 1 and Atmosphere Archive and Distribution System (LAADS) [45]. AOD data were available for Terra and Aqua satellites. There were two MODIS AOD observations per day, one for each satellite. Terra satellite had a late morning observation time, while the Aqua satellite had an early afternoon observation time. MODIS AOD data were re-mapped from NASA’s 10 km^2^ native grid system to CMAQ’s native 12 km^2^ grid system before the AOD data were uploaded in the upgraded HBM. Additional AOD dataset preparation details, including the technique used to estimate surface PM_2.5_ concentrations from the AOD data, have been published [16,46,47].

The HBM runs for New York City and Baltimore produced two AOD-estimated PM_2.5_ datasets: one AOD dataset with missing observations, and another dataset where the missing observations were replaced with kriged values (ordinary kriging). The original HBM required separating and sequencing the Bayesian computations into discrete hierarchical steps and then modeling at each level, before combining PM_2.5_ monitor measurements with CMAQ model predictions [48,49]. The original HBM was modified to process input surfaces with missing data and simultaneously combine multiple datasets [46]. This latter HBM upgraded feature was “critical” to permit the incorporation of AOD-PM_2.5_ input surfaces, in addition to PM_2.5_ monitor concentration readings and CMAQ PM_2.5_ model predictions.

### 2.3. AOD-PM_2.5_ Fused Surfaces

The upgraded HBM was used to produce PMB and the four experimental AOD-PM_2.5_ concentration surfaces, at the same time and utilizing the same statistical procedures, for both New York City [16] and Baltimore: (1) PMB statistically fused PM_2.5_ monitor concentration measurements with CMAQ PM_2.5_ model predictions. (2) PMC, the first AOD-PM_2.5_ surface, fused PM_2.5_ monitor concentration measurements with AOD-PM_2.5_ concentration values. This PMC included missing observations resulting from satellite recording failure or cloud cover interference in the recording column, extending from a satellite to the surface of the earth. (3) PMCK fused PM_2.5_ monitor concentration measurements with kriged AOD-PM_2.5_ concentration values. Kriging eliminated missing data from the pre-kriged PMC surface. (4) PMCQ included monitor PM_2.5_ concentration readings, CMAQ PM_2.5_ model predictions and PMC (not kriged). (5) PMCKQ incorporated monitor PM_2.5_ monitor concentration readings, CMAQ PM_2.5_ model estimates and PMCK (kriged). The shared properties of the four experimental AOD-PM_2.5_ fused surfaces made it possible to evaluate differences between AOD surfaces with and without missing observations (not kriged [PMC, PMCQ] vs. kriged [PMCK, PMCKQ]) and the absence and presence of CMAQ PM_2.5_ model predictions (absent [PMC, PMCK] vs. present [PMCQ, PMCKQ]). Table S2 (Supplementary Materials) summarizes additional information about PMB and the four experimental AOD-PM_2.5_-fused surfaces into columns that included PMB/AOD-PM_2.5_ surface name, surface description, and input surfaces used to produce each fused surface.

### 2.4. ED/IP Chronic Diseases

#### 2.4.1. Subjects

The 2004–2006 Maryland electronic ED visits and IP hospitalization confidential patient files, with one hospital record per patient per hospital encounter, were obtained from the Maryland Health Services Cost Review Commission (HSCRC) [50]. All hospitals in Maryland must report, by State statute, their ED visits/IP hospital events to the HSCRC quarterly. Available temporal variables only included years (2004–2006), quarters (winter, spring, summer, fall), and days of the week (Sunday through Saturday), but excluded month. Patient’s residential information was limited to 5-digit United States Postal Service Zone Improvement Plan (ZIP) codes (Maryland Department of Planning; MDP) [51]. Each patient record contained demographic variables, primary and secondary diagnoses, entered as International Classification of Diseases, Ninth Revision, Clinical Modification (ICD-9-CM) billing codes [52]. The ICD-9-CM codes were used to select asthma (493), myocardial infarction (MI, 410) and heart failure (HF, 428), as primary discharge diagnoses, and the co-morbid conditions of diabetes mellitus (250), hypertension (401), and atherosclerosis (414, 440), as secondary diagnoses. The Maryland Department of Health (MDH) Institutional Review Board approved this Baltimore AOD-PM_2.5_ data linkage and analysis study.

#### 2.4.2. Cases–Controls

The case-crossover design was used to form three different controls for each case [53–57]. Controls differed from cases only in the assigned exposure period. A case had one quarterly exposure value. Each control had one monthly mean exposure value for each of the four quarters: the first control had monthly mean exposure data for January, April, July, and October; the second control had monthly mean exposure data for February, May, August, and November; the third control combined monthly mean exposure data for March, June, August, and December. Each monthly mean exposure data represented a different calendar quarter (January–March, winter; April–June, spring; July–September, summer; October–December, fall). For each of the three controls, the selected four monthly mean exposures values were averaged, and the overall mean was used as the annual background exposure estimate.

Maclure’s [56] case-crossover design paper proposed using cases as their own controls but assigning a different exposure period to the same cases that were also used as controls. Since the same cases are used as controls, the cases and controls should not differ from each other on patient attributes such as gender, age, race, and insurance coverage. This is what we did in this study, because the month temporal variable was not available in the confidential hospital files obtained from the Maryland HSCRC, but was available in the New York City study [16].

The overall average for four months, with each month serving as a proxy for each of the four quarters in one year, provided an estimate of annual (background) exposure level. Since a different month was used to represent each quarter in each of the three controls, the exposure assignment algorithm used in this study is “functionally” equivalent to the case-crossover bidirectional design used in the New York City study [16,53]. In this study, cases with 1st and 2nd quarter exposure values always preceded all three controls because their mean monthly ranks (Q_1_ = 2.0, Q_2_ = 5.0) were always numerically smaller than the mean monthly ranks of all three controls (1st = 5.5, 2nd = 6.5, 3rd = 7.5). However, when cases had 3rd and 4th quarter exposure values they always came after all three controls, because their mean monthly ranks (Q_3_ = 8.0, Q_4_ = 11.0) were always numerically larger than the mean monthly ranks of all three controls.

### 2.5. Confounders

#### 2.5.1. Co-Morbid Conditions (Diabetes, Hypertension, Atherosclerosis)

Diabetes, hypertension, and atherosclerosis, when present in a patient’s hospital record, can synergistically contribute to the occurrence of a patient’s ED visit or IP hospitalization with a discharge diagnosis of asthma, MI, or HF. Diabetes mellitus, hypertension, and atherosclerosis by themselves have been shown to lead to an ED visit or IP hospitalization [13,16,58–62].

#### 2.5.2. Apparent Temperature (AT and AT^2^)

Ambient temperature, relative humidity, and wind speed can contribute to the occurrence of respiratory-cardiovascular chronic disease ED visits or IP hospitalizations. AT is one summary variable that includes ambient temperature, relative humidity, and wind speed. All three weather parameters were obtained from the CMAQ model [25] and made available to the Baltimore investigators by one of the co-authors (ESH). AT was computed using the formula reported on the National Oceanic and Atmospheric Administration website (NOAA) [16,63,64]. AT^2^ was computed as the product of AT*AT, once AT was available. Both AT and AT^2^ have been shown to influence respiratory-cardiovascular ED visits and IP hospitalizations, even in the absence of elevated ambient PM_2.5_ [13]. AT and AT^2^ values were computed for each CMAQ grid cell, which were stratified by year, month, and day.

#### 2.5.3. Pollen

Recent publications have implicated ambient pollen levels in respiratory-cardiovascular chronic disease ED visits/IP hospitalizations [65–67]. The single pollen counting station in Baltimore County, Maryland, provided the multi-year pollen readings. They were used as proxy measures for ambient pollen levels in the Baltimore study area [68].

#### 2.5.4. Holidays

Each major holiday included the day after each holiday. The dates of recognized holidays were obtained from the U.S. Office of Personnel Management website (OPM) [69]. Each holiday was combined in a single annual dummy variable and coded as 1 (holiday and day after) or 0 (no holiday). The holiday dummy variables were entered in each of the three annual data files for 2004–2006.

#### 2.5.5. Snowstorms

Each snowstorm was coded as a dummy variable, 1 (snowstorm) or 0 (no snowstorm), for each of the three years separately (National Centers for Environmental Information (NCEI) [70]. The snowstorm variable was a proxy for physical exertion during winter snow removal and its precipitation of a cardiovascular event, such as MI taking place, followed by an ED visit or IP hospitalization.

### 2.6. Effect Modifiers

#### 2.6.1. Poverty and Population Density

Population density and poverty co-occur more often in urban areas compared to rural locations. Brochu and colleagues [71] found a significant inverse association between economic resources and ambient PM_2.5_ concentration levels. Bell and Ebisu [72] found a higher poverty percent in census tracts with ambient air monitors compared to census tracts without monitors. Both are associated with barriers to healthcare access, e.g., fewer hospitals in rural than urban areas, and more frequent use of ED medical services by persons with fewer economic resources and no health insurance. Maryland Zip Code Tabulation Area (ZCTA) poverty percent and population density (subsequently converted to the log_10_ scale) data were obtained from the US Census Bureau website (USCB) [73].

#### 2.6.2. Season

Although Weber et al. [16] did not find warm–cold season differences, others have found season differences in the contribution of PM_2.5_ to respiratory-cardiovascular chronic disease hospital events [12,59,74–79]. Warm vs. cold season differences were evaluated in this study.

### 2.7. File Linkage

Given that the ED visit and IP hospitalization files included temporal variables for year (2004–2006), quarter (winter, spring, summer, fall), day of week (Sunday through Saturday) and spatial variables for residential five-digit ZIP codes, it was necessary to map these ZIP codes to CMAQ grids. It was also necessary to map ZCTA polygons for poverty percent and population density log_10_ measures to CMAQ grids.

ZIP code and ZCTA latitude–longitude centroid coordinates were entered in a Geographic Information System (GIS), which included a multi-layered map of Baltimore City/Maryland Counties and a 11 (south to north) by 9 (west to east) 12 km^2^ CMAQ grid map layer of the Baltimore study area, to develop a ZIP code-ZCTA-CMAQ 12 km^2^ grid polygon correspondence file. This assignment was done for each year separately [51,73,80]. Latitude-longitude centroid coordinates of 5-digit ZIP codes and ZCTAs were mapped to the interior area of a unique CMAQ 12 km^2^ grid cell. This ZIP code/ZCTA polygon assignment to CMAQ km^2^ grid cells was done for each of the three years separately, 2004–2006.

Base Statistical Analysis System (SAS) software, version 9.4, was used to link PMB and the four experimental AOD-PM_2.5_ concentration surfaces, de-identified ED/IP hospitalization records, confounders and effect modifiers by using the same assigned CMAQ grid identifier (1–99) and the temporal variables of year (3), quarter (4) and day of the week (7) [81].

#### 2.7.1. Case-Crossover Analyses

Each stratum included one case and three controls. Based on the time-space grouping variables of year (three), quarter (4), day of week (7), and spatial CMAQ 12 km^2^ grid cells (99), there were 8316 possible combinations of space–time locations that a case paired with three controls could be assigned. SAS/STAT Proportional Hazards Regression (PHREG) Procedure (Proc) was used to perform a regression analysis of stratified subpopulation’s “survival time” data. These conditional logistic regression analyses, based on the SAS/STAT PHREG Proc, use the Cox proportional hazards model, and the results explain the effect of time-dependent explanatory variables on survival times [82]. SAS/STAT PHREG Proc, version 14.3, and Base SAS, version 9.4, software programs were used to complete all conditional logistic regression analyses [57,81,82]. In these regression analyses, ties (for [censored] survival/failure times or [uncensored] event times under the Cox proportional hazards model) were set using the Breslow methodology [82,83].

#### 2.7.2. Statistical Analyses

The Chi Square test, in Base SAS, version 9.4, was used to evaluate case-control count data differences in age categories, gender, race, co-morbid conditions and health insurance [81]. By comparing the mean of one group with the lower and upper values for the 95% Confidence Interval (CI) of the reference group mean, it was possible to determine if the two means were significantly different from each other at *p* ≤ 0.05. A group mean was significantly different from the reference group mean if the value for the first comparison mean was either below the lower limit or above the upper limit of the 95% CI of the reference group mean (*p* ≤ 0.05). The Means Procedure in Base SAS, version 9.4, was used to compute means and 95% CIs for poverty percent and population density [81].

### 2.8. Conditional Logistic Regression Analyses

#### 2.8.1. Variable Selection

A multi-step variable evaluation procedure was used for all conditional logistic regression runs in all CMAQ grid cells, with and without ambient air monitors [57,84]. The starting statistical run only contained PMB, or one experimental AOD-PM_2.5_ concentration surface, and confounders (holidays, snowstorms, pollen, and AT and/or AT^2^). The index ED visit/IP hospitalization day was identified as lag day 0. The day before was lag day 1, and so on, through to lag day 4. Summary lag day measures for individual lag days of 0 through 4 were also computed. Summary lag days were obtained by taking the average of the included individual lag days. To illustrate, summary lag days 0 and 1, displayed as lag days 01. Lag days 2–4 were referenced as lag days 24. Lag days of 0 through 4 were named lag days 04. The AT^2^ was also entered on lag days 0, 1, 01 and 04. Effect modifiers were evaluated in separate conditional logistic regression runs (diabetes mellitus, hypertension, atherosclerosis; gender, age, race; health insurance, poverty, population density; and, season). An effect modifier was retained and included in the subsequent variable assessment runs if it had a computed probability value of *p* ≤ 0.20.

#### 2.8.2. Variable Evaluation of Effect Modifiers

This variable evaluation phase involved utilizing PMB or one of the four experimental AOD-PM_2.5_ concentration surfaces, and retained confounders on lag days of 0–4, 01, 24, and 04, in all grids and in grids with and without monitors. AT^2^ was added on lag days of 0, 1, 01 and 04. Each retained effect modifier was evaluated in a separate run. Each effect modifier with *p* ≤ 0.20 was evaluated in the final conditional logistic regression runs.

#### 2.8.3. Final Models

A stepwise procedure, with a variable entry criterion of *p* ≤ 0.20 and variable stay criterion of *p* ≤ 0.09, was used to identify the most parsimonious combination of confounders, effect modifiers for each PMB and the four experimental AOD-PM_2.5_ concentration surfaces in all grids, and in grids with and without monitors. The Akaike Information Criterion (AIC) statistic was also used to confirm the selection of the “best” conditional logistic regression runs, with lower AIC values representing a better model fit [84]. The null hypothesis was rejected when *p* ≤ 0.05 [85].

#### 2.8.4. Season and Monitor

Follow-up conditional logistic regression analyses evaluated the main factors of season (S), monitor (M) and the interaction term of S*M, for PMB and the four experimental AOD-PM_2.5_ concentration surfaces, at lag days of 0, 1 and 01, in all grids, and in grids with and without monitors, for ED asthma, IP asthma, IP MI, and IP HF, in separate analyses.

## 3. Results

### 3.1. Cases and Controls

Case-control group characteristics are in Table 1 (ED and IP asthma) and Table 2 (IP MI and IP HF). As stated previously, there were three controls for each case in each of the four groups. There were more ED asthma cases (11,723) than IP asthma cases (3376), with IP MI (4745) and IP HF (6919) between two asthma case groups. ED asthma cases were significantly younger than IP asthma cases (*p* ≤ 0.05). The 0–14 age category included 43.8% ED asthma cases but only 27.6% IP asthma cases. The 35+ age category contained 61.8% of the IP asthma cases but only 28.7% of the ED asthma cases. There was also a significant age category difference between IP HF cases and IP HF controls (*p* ≤ 0.05), with lower percentages for cases (18.5%) and controls (19.2%) in the 35–59 age category and higher percentages for cases (44.9%) and controls (46.0%) in the 76+ age category. Other significant differences were found between grids without monitors and grids with monitors for poverty percent and population density for all case and control groups (all *p’s* ≤ 0.05).

### 3.2. AOD-PM_2.5_ Concentration Surfaces

#### 3.2.1. PMB and AOD-PM_2.5_

Only the PMCK PM_2.5_ concentration three-year mean of 14.38 µg/m^3^ (95% CI = 14.31–14.44) was significantly higher than the PMB PM_2.5_ three-year mean of 14.19 (95% CI = 14.13–14.26) (*p* ≤ 0.05). The other three experimental AOD-PM_2.5_ concentration surface means (PMC = 13.66 (95% CI = 13.60–13.72), PMCQ = 13.79 (95% CI = 13.73–13.85), and PMCKQ = 13.91 (95% CI = 13.85–13.97) were significantly lower than the PMB mean (all *p’s* ≤ 0.05). The unexpected finding was confirmation that the Baltimore PMB and four experimental AOD-PM_2.5_ concentration surfaces were *significantly higher* than the New York City PMB and four experimental AOD-PM_2.5_ concentration surfaces (PMB = 10.02; PMC = 12.03, PMCK = 10.51; PMCQ = 10.09; PMCKQ = 12.91) (all *p’s* ≤ 0.05). Table S3 (Supplementary Materials) summarizes these and other descriptive statistics (percentiles) for PMB and the four experimental AOD-PM_2.5_-fused surfaces.

#### 3.2.2. Correlations between AOD-PM_2.5_ and PMB

The *r*^2^% statistic, representing the percentage of shared variance between PMB and another experimental AOD-PM_2.5_ concentration surface, was highest between PMB and PMCQ in all three grid conditions (both, 94.7%; with monitors, 97.4%; without monitors, 94.3%). This measure of shared variance was lowest between PMCK and PMB (both, 30.6%; with monitors, 62.1%; without monitors, 26.5%). The *r*^2^% difference (no monitors versus monitors) produced a similar ranking, with the smallest negative difference for PMCQ (−3.1%), and the largest for PMCK (−35.6%), followed by PMCKQ (−15.9%) and PMC (−32.4%) with intermediate values between the first two. Table S4 (Supplementary Materials) contains correlation analyses for PMB with each of the four experimental AOD-PM_2.5_-fused surfaces.

#### 3.2.3. PM_2.5_ Concentration Values

Three-year PM_2.5_ concentration means (95% CIs) were computed for all lag days, surfaces, and monitor grid conditions. All comparisons were between each AOD-PM_2.5_ concentration surface and PMB. PMCK PM_2.5_ concentration means were significantly higher than PMB PM_2.5_ concentration means in all grids and in grids without monitors for all lag days (all *p’s* ≤ 0.05). The monitor PMCK PM_2.5_ concentration surface was significantly lower than the monitor PMB PM_2.5_ concentration surface at all lag days (*p* ≤ 0.05). All other comparisons between PMC, PMCQ and PMCKQ PM_2.5_ concentration surfaces were also significantly lower than PMB for all lag days of 0–4, 01, 24 and 04 in all grids, in grids with monitors and in grids without monitors (all *p’s* ≤ 0.05). Supplementary Materials includes three-year PM_2.5_ concentration mean values for PMB and each of the four AOD-PM_2.5_ concentration surfaces on lag days 0–4, 01, 24 and 04 in all CMAQ grids (Tables S5 and S6), in grids with monitors (Tables S7 and S8) and in grids without monitors (Tables S9 and S10).

### 3.3. Conditional Logistic Regression

Significant conditional logistic regression analyses for the four health outcomes, three CMAQ grid conditions, and the four experimental AOD-PM_2.5_ concentration surfaces and PMB only occurred at lag days of 0, 1 and 01 (all *p’s* ≤ 0.01). Significant, but protective, population density (M) effect modifiers were found for ED asthma in all grids for PMB (lags of 01), PMCQ (lags of 0, 1 and 01) and PMCKQ (lags of 01) (all *p’s* ≤ 0.01). Significant, but protective, Season (S) effect modifiers occurred for ED asthma in all grids at lags of 01 (PMB, PMC, PMCK, PMCQ and PMCKQ) and for IP HF in all grids at lags of 01 (PMB, PMC, PMCK, PMCQ, PMCKQ) (all *p’s* ≤ 0.05).

Odds Ratios (ORs) and 95% Confidence Intervals (CIs) for the four health outcomes, under the three grid conditions, PMB and the four experimental AOD-PM_2.5_ concentration surfaces, are displayed graphically below, and separated into successive graphs by lag days of 0, 1 and 01. To determine if the AOD-PM_2.5_ concentration surface ORs differed from the PMB OR, each AOD-PM_2.5_ OR was compared to the PMB’s OR (95% CI). If the AOD-PM_2.5_ OR was either below or above the PMB’s 95% CI lower or upper limit, the outcome was significant (*p* ≤ 0.05).

#### 3.3.1. Lag Day 0 (Figure 2A–D)

**Figure 2.**
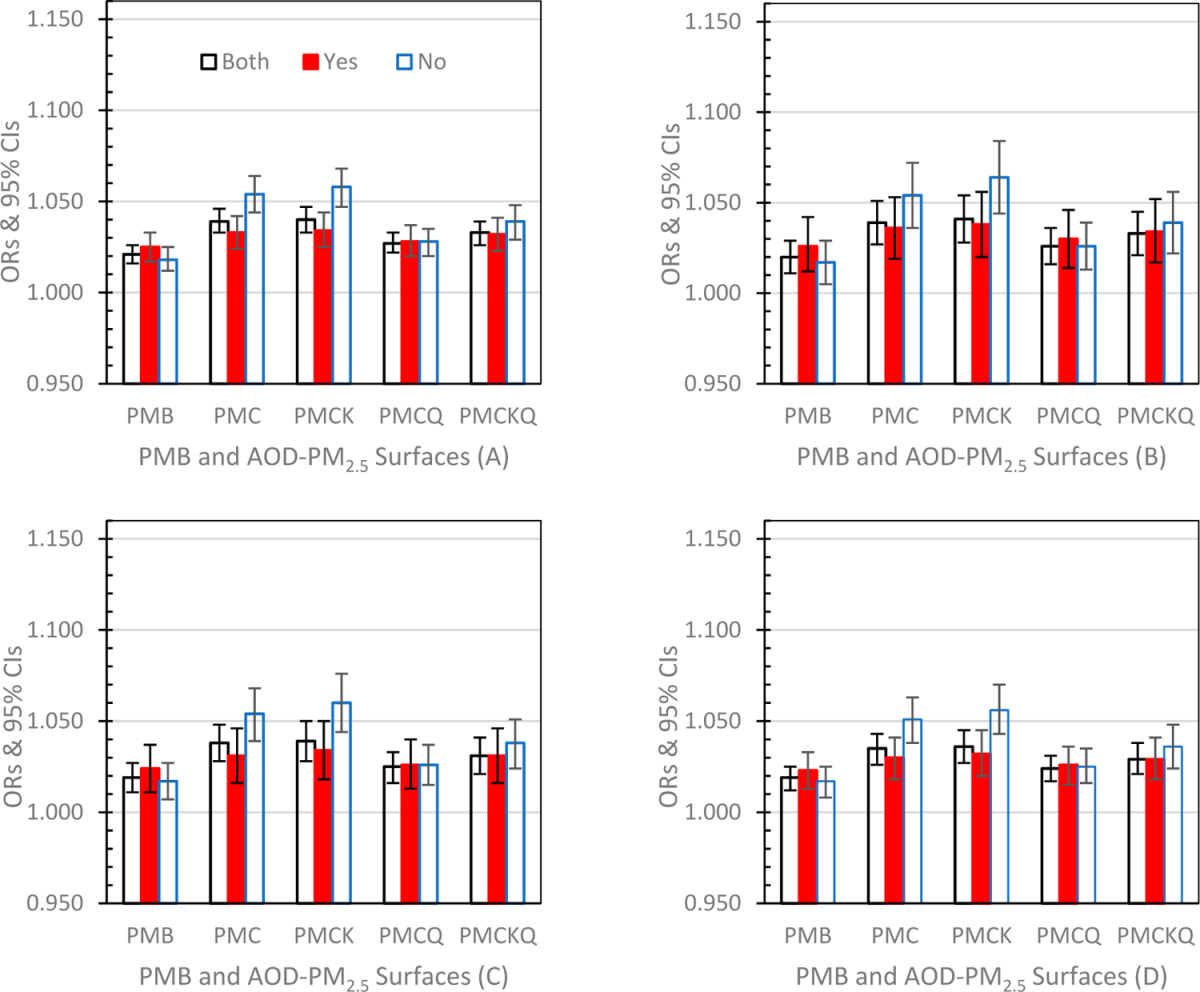
Odds Ratios (ORs) and 95% Confidence Intervals (CIs) for the four experimental aerosol optical depth (AOD)-particulate matter (PM)_2.5_ concentration surfaces and particulate matter baseline (PMB) under both grid conditions (Both), grids with monitors (Yes) and grids without monitors (No) at lag day 0: (**A**) ED asthma (top left panel), (**B**) IP asthma (top right panel), (**C**) IP MI (bottom left panel), and (**D**) IP HF (bottom right panel).

For ED asthma (Figure 2A) in all grids (Both) and in grids without monitors (No) each of the four AOD-PM_2.5_ ORs were significantly higher than the PMB OR (all *p’s* ≤ 0.05). However, in grids with monitors (Yes), only the PMCK OR was significantly higher than the PMB OR (*p* ≤ 0.05). For IP asthma (Figure 2B), IP MI (Figure 2C) and IP HF (Figure 2D) in all grids and in grids without monitors, only PMC, PMCK and PMCKQ ORs were significantly higher than PMB ORs (all *p’s* ≤ 0.05). For all four health outcomes, only PMC and PMCK had significantly higher ORs in the no monitor condition than the PMC and PMCK ORs in the monitor condition (all *p’s* ≤ 0.05).

#### 3.3.2. Lag Day 1 (Figure 3A–D)

**Figure 3.**
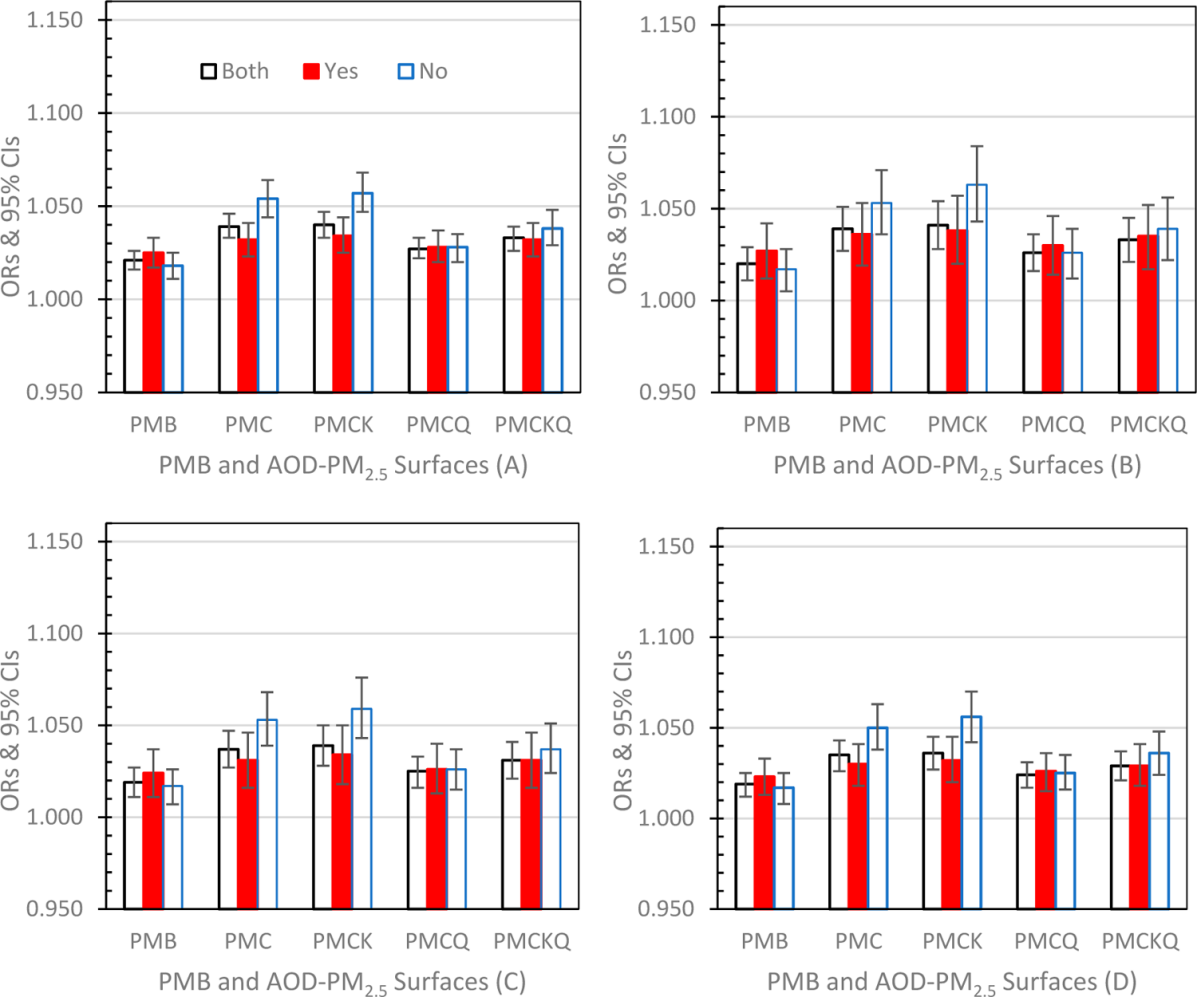
Odds Ratios (ORs) and 95% Confidence Intervals (CIs) for the four experimental AOD-PM_2.5_ concentration surfaces and PMB under both grid conditions (Both), grids with monitors (Yes) and grids without monitors (No) at lag day 1: (**A**) ED asthma (top left panel), (**B**) IP asthma (top right panel), (**C**)IP MI (bottom left panel) and (**D**) IP HF (bottom right panel).

The ED asthma (Figure 3A), IP asthma (Figure 3B), IP MI (Figure 3C) and IP HF (Figure 3D) OR results for all comparisons between each of the four AOD-PM_2.5_ concentration surfaces with the PMB ORs at lag 1 in all grids, and in grids without and with monitors were identical to the comparisons made on lag day 0 and described above (all *p’s* ≤ 0.05). No monitor versus monitor OR comparisons for all four health outcomes were also the same as those previously described above on lag day 0 (*p* ≤ 0.05).

#### 3.3.3. Lag Day 01 (Figure 4A–D)

**Figure 4.**
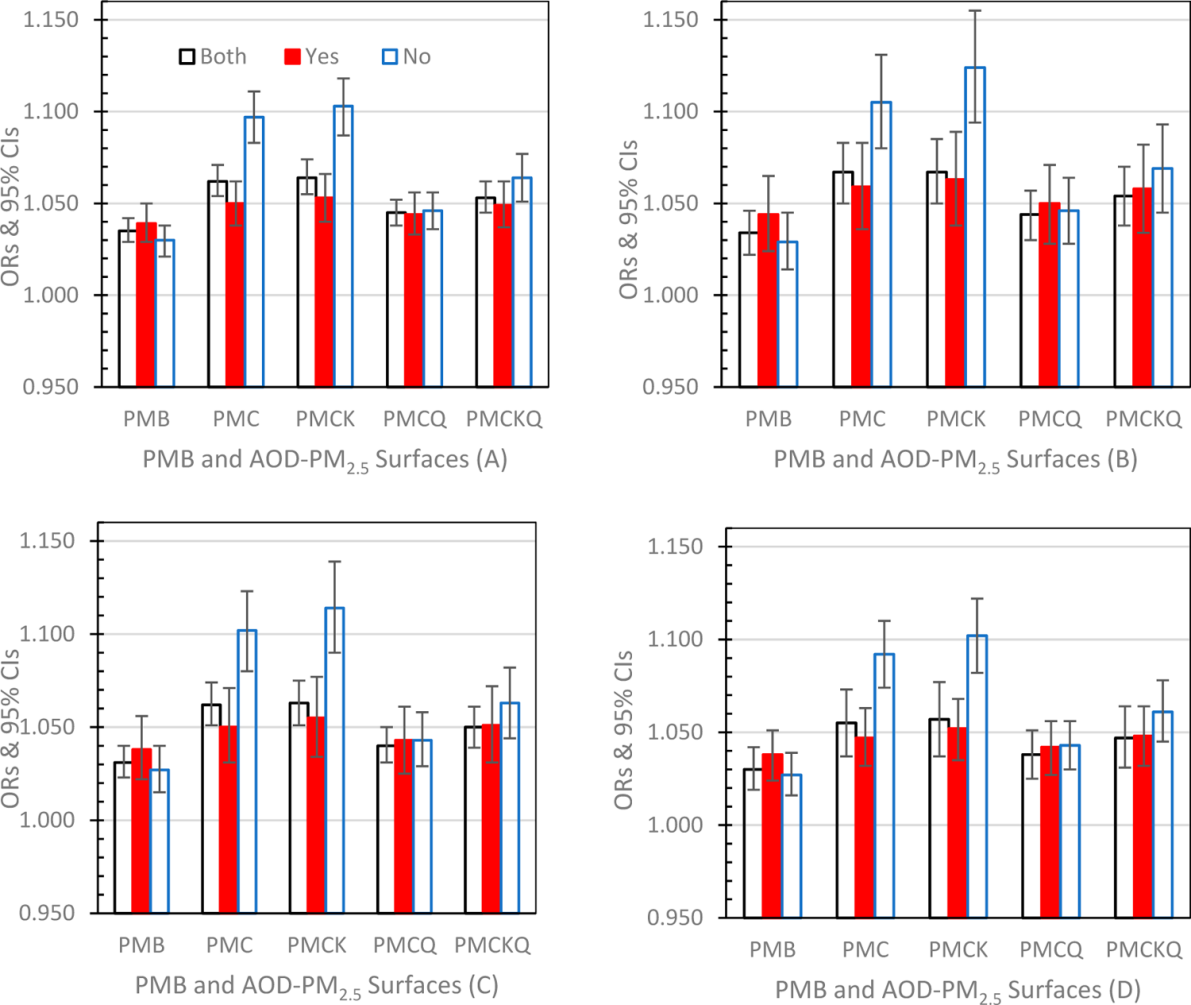
Odds Ratios (ORs) and 95% Confidence Intervals (CIs) for the four experimental AOD-PM_2.5_ concentration surfaces and PMB under both grid conditions (Both), grids with monitors (Yes) and grids without monitors (No) at lag days 01: (**A**) ED asthma (top left panel), (**B**) IP asthma (top right panel), (**C)** IP MI (bottom left panel), and (**D**) IP HF (bottom right panel).

ED asthma (Figure 4A), IP asthma (Figure 4B), IP MI (Figure 4C) and IP HF (Figure 4D) comparisons between AOD-PM_2.5_ ORs and PMB ORs at lag days 01 were the same as the comparisons at lag day 0 and lag day 1 and described above (all *p’s* ≤ 0.05). For IP asthma, IP MI, and IP HF in the no monitor grid condition PMCQ ORs were significantly higher than the PMB ORs (all *p’s* ≤ 0.05). In addition, in grids with monitors for ED asthma and IP HF PMCK ORs were significantly higher than the PMB ORs (both *p’s* ≤ 0.05). The no monitor OR versus the monitor OR comparisons for PMC and PMCK were the same as those previously described for lag days 0 and 1 (all *p’s* ≤ 0.05). In addition, for ED asthma the no monitor PMCKQ OR was significantly higher than the monitor PMCKQ OR (*p* ≤ 0.05).

### 3.4. Lag Day and Monitor

**Figure 5.**
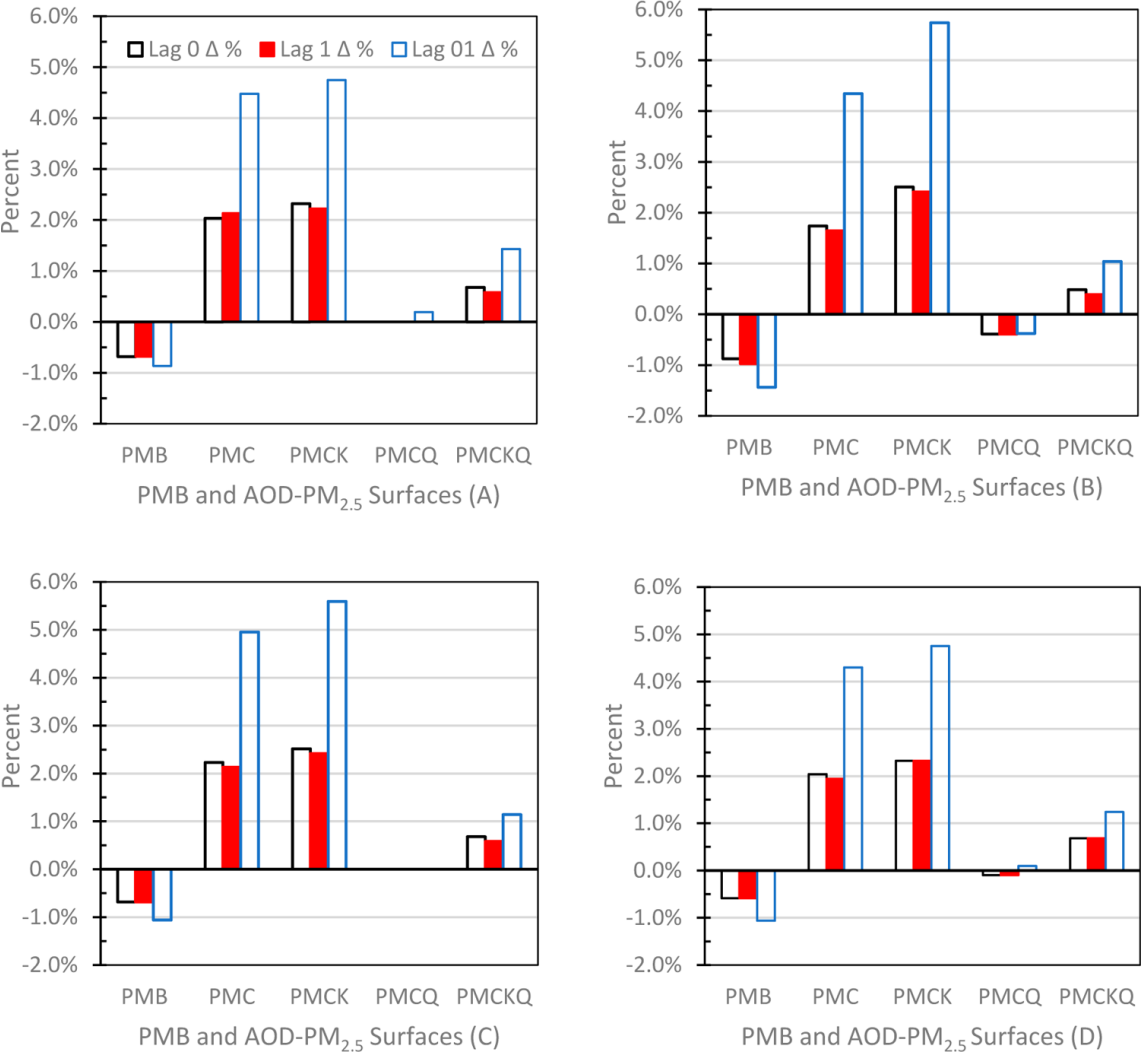
Percent change between no monitor and monitor Odds Ratios (ORs) for the four experimental AOD-PM_2.5_ concentration surfaces and PMB at lag days of 0, 1 and 01: (**A**) ED asthma (top left panel), (**B**) IP asthma (top right panel), (**C**) IP MI (bottom left panel), and (**D**) IP HF (bottom right panel).

Further evidence supporting the robustness of PMC and PMCK concentration surfaces, especially in grids without monitors, is summarized in Figure 5A–D. Percentage change in ORs in grids without monitors versus ORs in grids with monitors at lag days of 0, 1 and 01 demonstrate the same pattern for ED asthma (A), IP asthma (B), IP MI (C) and HF (D): (1) PMC and PMCK concentration surfaces had positive percent change values, with the largest increase always occurring at lag days 01. (2) All PMB percent change values were negative. (3) PMCQ results resembled most the PMB outcomes, with negative percent change values at lag days of 0, 1 and 01 for IP asthma (B) and at lag days of 0 and 1 for IF HF (D), or close to 0% change for IP MI (C) and ED asthma at lag days of 0 and 1 (A). (4) In all four panels, the PMCKQ surface had positive percent change values, thereby suggesting that kriging partly reversed the percent decrease due to the inclusion of CMAQ.

### 3.5. Lag Day and Season

**Figure 6.**
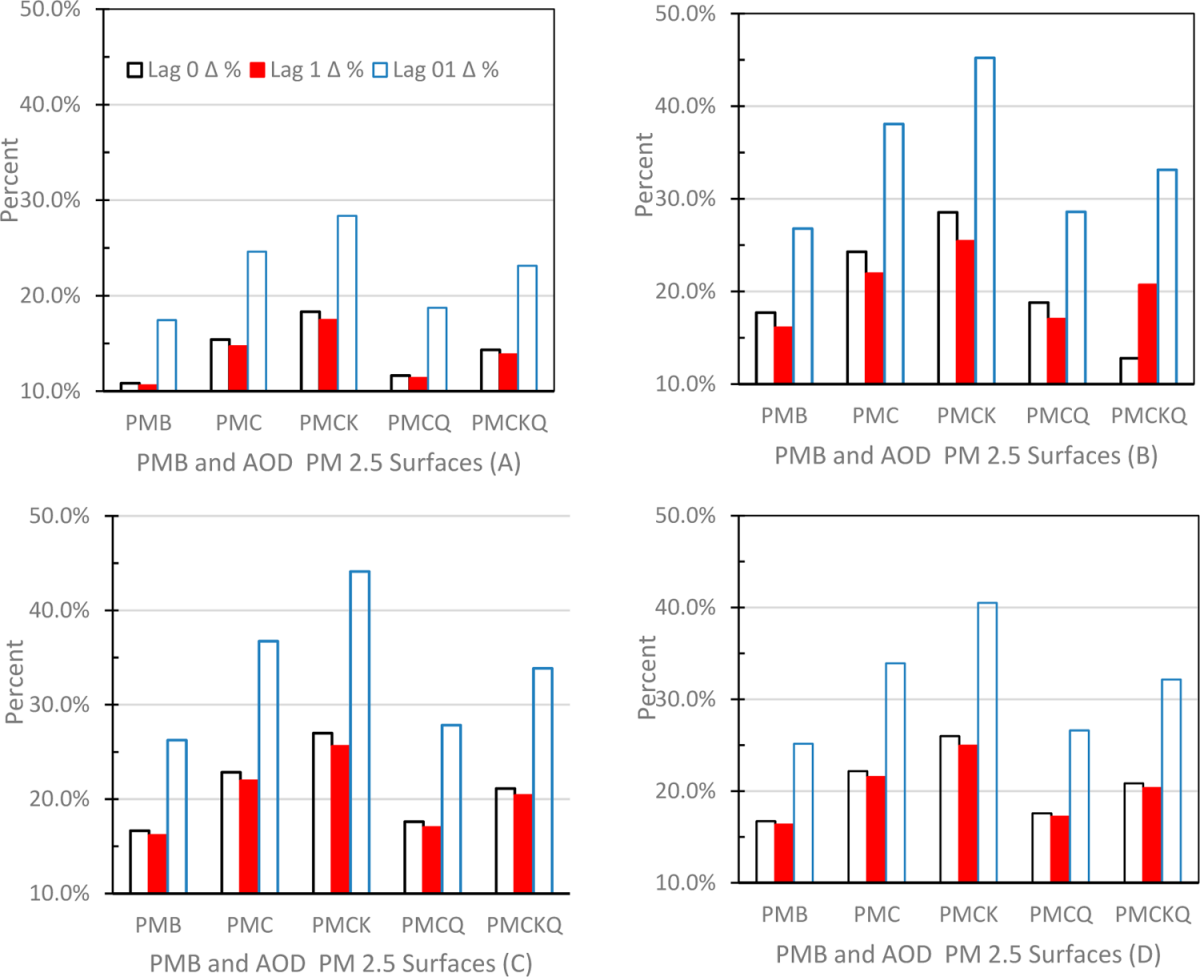
Percent change between warm and cold season Odds Ratios (ORs) for PMB and the four experimental aerosol optical depth (AOD)-PM_2.5_ concentration surfaces at lag days of 0, 1 and 01: (**A**) ED asthma (top left panel), (**B**) IP asthma (top right panel), (**C**) IP MI (bottom left panel) and (**D**) IP HF (bottom right panel).

Results of follow-up conditional logistic regression analyses evaluating warm–cold season (S) differences at lag days of 0, 1 and 01 on PMB and the four AOD PM_2.5_ concentration surfaces support these conclusions: (1) only the cold season ORs were protective, e.g., below 1.000. (2) All warm season ORs were significantly higher than cold season ORs (all *p’s* ≤ 0.05). (3) During the warm season, only ED asthma, IP MI, and IP HF PMCK ORs were significantly higher than PMB ORs (all *p’s* ≤ 0.05). Figure 6 below shows the percent change between warm season and cold season ORs for the four AOD-PM_2.5_ concentration surfaces and PMB on lag days of 0, 1 and 01 for the four hospital-based health outcomes. For all four health outcomes the largest percent increase occurred at lag day of 01. Figures S1–S3 (Supplementary Materials) display warm–cold season ORs for the four AOD-PM_2.5_ experimental surfaces and PMB for ED asthma, IP asthma, IP MI, and IP HF at lag days of 0, 1 and 01, respectively.

## 4. Discussion

The shared objectives of the Baltimore and New York City [16] studies were to evaluate the differential contribution of the four experimental AOD-PM_2.5_ concentration surfaces relative to the current baseline, PMB, on ED asthma, IP asthma, IP MI and HF hospitalizations. Our expectation was to find the most improvement in the new experimental AOD-PM_2.5_ concentration surface, which included PMC (not kriged or kriged) fused with PMB (monitor PM_2.5_ and CMAQ PM_2.5_ model estimates). The New York City study did not find differences between PMB and the four AOD-PM_2.5_ concentration surfaces. Contrary to our expectation, these Baltimore study results suggest that PMC and PMCK, and not PMCQ or PMCKQ, are better estimates of ambient PM_2.5_, especially in grids without monitors.

Although the same upgraded HBM was used to generate the PMB and the four AOD-PM_2.5_ concentration surfaces for Baltimore and New York City, all five Baltimore surfaces had *significantly higher* three-year mean PM_2.5_ concentration values than the New York City surfaces. Since both the Baltimore and New York City study sites analyzed the same asthma, MI and HF chronic diseases, used the case-crossover design to create three controls for each case, and used the same SAS conditional logistic regression procedure to analyze the linked exposure–health outcome files, the only remaining *major* difference was the significantly elevated ambient PM_2.5_ levels in Baltimore compared to New York City.

The three methodological differences between the two study sites included: (1) more asthma, MI, and HF cases (and associated controls) in the New York City study than in the Baltimore study. (2) Completion of separate ED asthma and IP asthma conditional logistic regression analyses in the Baltimore study than in the New York City study, because ED asthma cases (and associated controls) were significantly younger than the IP asthma cases (and associated controls). (3) In the Baltimore study, the three controls were formed by assigning a different exposure period to the cases that were also used as controls [56], while the New York City study selected the three controls within a 28 day strata. Both test site replications attained the same case–crossover endpoint, since each case was preceded or followed by at least one control—the definition of a bidirectional case-crossover design [53,55]. It is unlikely that these methodological differences would be more important than the fact that the Baltimore study three-year mean PMB and four AOD-PM_2.5_ concentration surfaces had significantly higher concentration values than the New York City study.

This Baltimore study also found differences in the contribution of PMB and the four experimental AOD-PM_2.5_ concentration surfaces to asthma ED visits and IP asthma, IP MI, and IP HF hospitalizations in grids without monitors. In grids without monitors, PMC, PMCK and PMCKQ ORs were significantly higher than PMB ORs at lag days of 0, 1 and 01, for all four ED/IP respiratory-cardiovascular chronic diseases. In grids with monitors, only ED asthma PMCK ORs were significantly higher than PMB ORs for all three lag days of 0, 1 and 01. A similar outcome occurred for IP HF, but only on lag days of 01. Additional no monitor versus monitor analyses showed that only PMC and PMCK had significantly higher ORs in grids without monitors than in grids with monitors at all three lag days of 0, 1 and 01 for all four respiratory-cardiovascular chronic disease hospital events. The only exception was for IP asthma at lag day of 1, where the PMC no monitor versus monitor comparison was not significant.

Since two AOD-PM_2.5_ surfaces included CMAQ PM_2.5_ model estimates, PMCQ (PMC not kriged) and PMCKQ (PMCK kriged), we now know that a bias is introduced by the addition of CMAQ PM_2.5_ model estimates to the experimental AOD-PM_2.5_ concentration surfaces. This bias was expressed by shifting the PM_2.5_ concentration values of this surface, PMCQ, toward the PMB PM_2.5_ concentration values. PMCKQ resembled PMB less, a reversal that can be attributed to kriging.

Results from this Baltimore study support these conclusions: Firstly, only PMCQ ORs in all grids at lag days of 0, 1 and 01 and in grids without monitors at lag days of 0 and 1, were not significantly different from PMC ORs for IP asthma, IP MI and IP HF. Secondly, no monitor PMCQ, PMCKQ and PMB ORs were not significantly different from monitor PMCQ, PMCKQ and PMB ORs at lag days 0, 1 and 01 for ED asthma, IP asthma, IP MI, and IP HF. Thirdly, the difference in the *r*^2^% statistic between PMCQ and PMB in grids with monitors (97.4%) and grids without monitors (94.3%) was negatively smaller (−3.1%) than it was for PMCKQ (−15.9%), PMC (−32.4%) and PMCK (−35.6%). These results suggest that PMC and PMCK could be used as a replacement for CMAQ PM_2.5_ model estimates in an upgraded HBM-generated PMB.

We also found significant season effects in the Baltimore study, which were not found in the New York City study [16]. In this study only warm season ORs were greater than 1.000, while cold season ORs were below 1.000, and therefore protective *for those affected cases*. Warm season PMCK ORs at lag days of 01 were significantly higher than cold season PMCK ORs for ED asthma, IP MI, and IP HF. Some published studies have reported significant warm season effects [12,76,78], while other publications have reported significant cold season effects [74,75,77,79]. Kuo and colleagues [78] found an association between PM_2.5_ concentration level and asthma hospitalization in children in the fall season. One interpretation of warm season effects, based on the positive results of this study and the non-significant outcome in the New York City study [16], is higher ambient PM_2.5_ concentration levels during the warm season compared to the cold season [8].

Surprisingly, there are only *two* publications on PM_2.5_ concentration levels and respiratory-cardiovascular hospitalizations in urban Baltimore [14,15], and *no* published papers in rural areas. This study demonstrated, for the first time, that PM_2.5_ concentration level does contribute to respiratory-cardiovascular hospital events, ED visits and inpatient stays, both in Baltimore City, with its higher population density and poverty, and in other study area grids that lacked ambient air monitors, and had lower population density and poverty. By analyzing grids without monitors, we were able to demonstrate that in these rural areas PMC and PMCK were associated with ED visits and IP hospitalizations of respiratory-cardiovascular chronic conditions. Others have also found associations between ambient PM_2.5_ concentration levels and respiratory-cardiovascular hospital events in rural areas [6,71], but due to course PM [86]. Strickland and associates [10] reported an association between PM_2.5_ concentration and ED asthma visits, but no change due to levels of urbanicity.

There are methodological limitations and strengths to the Baltimore study. First, these results were based on 2004–2006 ED visits and IP hospitalizations that, in 2020, are more than a decade old. Baltimore ambient PM_2.5_ levels were higher in 2004–2006 than in 2020. Replicating the Baltimore study with more current HBM-generated PMB and experimental AOD-PM_2.5_ concentration surfaces and ED/IP respiratory-cardiovascular chronic diseases would go a long way to confirm the generalizability of these exploratory results. A second limitation is the smaller Baltimore study area that only included 99 CMAQ 12 km^2^ grids. A replication of this study should enlarge the CMAQ grid study area to contain the entire state of Maryland.

Methodological strengths include the inclusion of confounders in all conditional logistic regression analyses (apparent temperature, snow storms, major holidays, pollen apparent temperature), and the evaluation of effect modifiers (poverty, population density, season). A correspondence file was used to assign ZIP code and ZCTA polygons to a *single* CMAQ grid. This procedure was used to *minimize* differences in polygon shapes found between ZIP codes (United States Postal Service) and ZCTAs (United States Census Bureau), and to uniformly complete all analyses by only using CMAQ grids. Evaluation of conditional logistic regression runs in all three CMAQ grid conditions, all grids, and in grids with and without monitors was another methodological strength of this study.

Within this decade, AOD readings with a grid resolution of 10, 5, 2, 1 and <1 km^2^ have become easier to obtain and use [27–29,31,33–36]. Kumar and colleagues evaluated NASA MODIS Level 1 and 2 in grid dimensions of 10, 5, and 2 km^2^ [33,34]. They concluded that AOD in 2 km^2^ grids provided greater resolution than AOD in either 10 km^2^ or 5 km^2^ grids [34]. The major advantages of AOD data in 2 km^2^ grids are: (1) higher correlation with on-the-ground ambient PM_2.5_ monitors; and, (2) a decrease in lost data due to cloud interference. Another advantage is related to “scale” effects. The higher resolution found in smaller grids permits more *precise* AOD PM_2.5_ measurements of ambient PM_2.5_ concentrations than in larger grids [87].

Another benefit of using AOD in <10 km^2^ grids is the possibility of replacing ZIP-code-aggregated health data with address-level health data [23,30,88–91]. Patient privacy issues require obtaining the patient’s written consent before using a person’s ED visit or IP hospitalization record in an environmental health epidemiologic study. The dual benefits of having available AOD data in <10 km^2^ grids with address-level data include the possibility of evaluating the contribution of ambient PM_2.5_ to ED visits and IP hospitalizations for respiratory-chronic diseases in both urban and rural areas, and utilizing remote sensing data to better understand shared pathophysiological mechanisms responsible for the occurrence of asthma, MI and HF events, and the development and testing of newer population-based intervention efforts.

## 5. Conclusions

If these Baltimore experimental AOD-PM_2.5_ concentration surface results are confirmed, then a shift in using AOD-PM_2.5_ concentrations readings in place of CMAQ PM_2.5_ model predictions in the PMB may be warranted, particularly in light of the greater temporal (every 5 min during daylight hours) and spatial (2 km^2^) data now available from geostationary satellites such as GOES-16 and GOES-17.

## Supplementary Material

supplementary

## Figures and Tables

**Table 1.** Demographics for ED Asthma and IP Asthma Cases and Controls in the Baltimore Study.

Variables ^1–2^	ED Asthma Cases	ED Asthma Controls	IP Asthma Cases	IP Asthma Controls
**Total**	11,723 (100)	35,533 (100)	3376 (100)	10,139 (100)
Age Category ^3–4^ 0–14 years	5131 (43.8) ^†^	15,492 (43.6) ^†^	930 (27.6)	2791 (27.5)
15–34 years	3223 (27.5)	9765 (27.5)	358 (10.6)	1080 (10.6)
≥35 years	3369 (28.7)	10,276 (28.9)	2088 (61.8)	6268 (61.8)
Gender—Female	6093 (52.0)	18,489 (52.0)	2125 (62.9)	6388 (63.0)
Male	5628 (48.0)	17,041 (48.0)	1251 (37.1)	3751 (37.0)
Race—Black	5618 (48.1)	17,078 (48.3)	1130 (33.5)	3380 (33.4)
Other	749 (6.4)	2311(6.5)	164 (4.9)	505 (5.0)
White	5305 (45.4)	15,989 (45.2)	2076 (61.6)	6236 (61.6)
Atherosclerosis—No	11675 (99.6)	35,387 (99.6)	3055 (90.5)	9176 (90.5)
Yes	48 (0.4)	146 (0.4)	321 (9.5)	963 (9.5)
Diabetes—No	11458 (97.7)	34,730 (97.7)	2842 (84.2)	8557 (84.4)
Yes	265 (2.3)	803 (2.3)	534 (15.8)	1582 (15.6)
Hypertension—No	11,111 (94.8)	33,650 (94.7)	2316 (68.6)	6950 (68.6)
Yes	612 (5.2)	1883 (5.3)	1060 (31.4)	3189 (31.4)
Insurance—No	2099 (17.9)	6409 (18.1)	207 (6.1)	623 (6.2)
Yes	9606 (82.1)	29,070 (81.9)	3164 (93.9)	9501 (93.8)
Poverty ^5^	9.6 (9.5–9.7)	9.6 (9.5–9.7)	9.4 (9.2–9.6)	9.4 (9.2–9.5)
Monitor—No	6.3 (6.3–6.4) *	6.4 (6.3–6.4) *	6.3 (6.2–6.5) *	6.3 (6.3–6.4) *
Monitor—Yes	13.7 (13.5–3.9)	13.7 (13.6–13.8)	13.6 (13.3–13.9)	13.5 (13.4–13.7)
Population (Log_10_) ^6^	3.3 (3.3–3.3)	3.3 (3.3–3.3)	3.2 (3.2–3.2)	3.2 (3.2–3.2)
Monitor—No	3.1 (3.1–3.1) *	3.1 (3.1–3.1) *	2.9 (2.9–2.9) *	2.9 (2.9–2.9) *
Monitor—Yes	3.6 (3.6–3.6)	3.6 (3.6–3.6)	3.5 (3.5–3.6)	3.5 (3.5–3.6)

1 Each column displays total observations (n) and percent (%) for emergency department (ED)/inpatient (IP) asthma case-control groups.

2 Significance was evaluated with the Chi Square test:

* = *p* ≤ 0.05;

† = *p* ≤ 0.01.

3 Significant age group differences between ED asthma cases and IP asthma cases, *p* ≤ 0.01.

4 Significant age group differences between ED asthma controls and IP asthma controls, *p* ≤ 0.01.

5 Significant differences between no monitor versus monitor within poverty, *p* ≤ 0.05.

6 Significant differences between no monitor versus monitor within population density (Population, L_10_), *p* ≤ 0.05.

**Table 2.** Demographics for IP Myocardial Infarction (MI) and IP Heart Failure (HF) Cases and Controls in the Baltimore study.

Variables ^1–2^	IP MI Cases	IP MI Controls	IP HF Cases	IP HF Controls
**Total**	4745 (100)	14276 (100)	6919 (100)	20,427 (100)
Age Category ^3–4^ 35–59 years	1477 (31.1) ^†^	4462 (31.3) ^†^	1279 (18.5) *	3921 (19.2)
60–75 years	1638 (34.5)	4907 (34.4)	2531 (36.6)	7119 (34.8)
≥76 years	1630 (34.4)	4907 (34.4)	3109 (44.9)	9387 (46.0)
Gender—Female	2041 (42.6)	6155 (42.7)	3559 (52.1)	10,808 (52.2)
Male	2749 (57.4)	8256 (57.3)	3267 (47.9)	9884 (47.8)
Race-Black	633 (13.2)	1913 (13.3)	1737 (25.5)	5292 (25.6)
Other	214 (4.5)	634 (4.4)	191 (2.8)	602 (2.9)
White	3937 (82.3)	11,843 (82.3)	4892 (71.7)	14,780 (71.5)
Atherosclerosis—No	1716 (35.8)	5163 (35.8)	3554 (52.1)	10,777 (52.1)
Yes	3074 (64.2)	9248 (64.2)	3272 (47.9)	9915 (47.9)
Diabetes—No	3363 (70.2)	10,101 (70.1)	3950 (57.9)	11,948 (57.7)
Yes	1427 (29.8)	4310 (29.9)	2876 (42.1)	8744 (42.3)
Hypertension—No	2511 (52.4)	7499 (52.0)	4022 (58.9)	12197 (59.0)
Yes	2279 (47.6)	6912 (48.0)	2804 (41.1)	8495 (41.1)
Insurance—No	149 (3.1)	443 (3.1)	111 (1.6)	324 (1.6)
Yes	4637 (96.9)	13,956 (96.9)	6710 (98.4)	20,356 (98.4)
Poverty ^5^	8.3(8.2–8.4)	8.4 (8.3–8.4)	9.1 (9.0–9.2)	9.2 (9.1–9.2)
Monitor—No	6.0 (5.9–6.1) *	6.0 (6.0–6.1) *	6.4 (6.3–6.5) *	6.4 (6.3–6.4) *
Monitor—Yes	12.2 (11.9–12.4)	12.3 (12.2–12.4)	2.8 (12.6–13.0)	12.8 (12.7–12.9)
Population (Log_10_)^6^	3.1 (3.1–3.1)	3.1 (3.1–3.1)	3.2 (3.2–3.2)	3.2 (3.2–3.2)
Monitor—No	2.8 (2.8–2.8) *	2.8 (2.8–2.8) *	2.9 (2.9–2.9) *	2.9 (2.9–2.9) *
Yes	3.6 (3.5–3.6)	3.6 (3.5–3.6)	3.6 (3.6–3.6)	3.6 (3.6–3.6)

1 Each column displays total observations (n) and percent (%) for IP MI and IP HF case-control groups.

2 Significance evaluated with the Chi Square test:

* = *p* ≤ 0.05;

† = *p* ≤ 0.01.

3 Significant age group difference between IP HF cases and controls, *p* ≤ 0.05.

4 Significant difference between no monitor versus monitor within poverty, *p* ≤ 0.05.

5 Significant difference between no monitor versus monitor within population density (Population, L_10_), *p* ≤ 0.05.

6 Significant differences between no monitor versus monitor within population density (Population, L_10_), *p* ≤ 0.05.

## Abbreviations

AIC: Akaike Information Criterion
AOD: Aerosol Optical Depth
AQS: Air Quality System
AT: Apparent Temperature
AT^2^: Product of AT, AT*AT
CDC: U.S. Centers for Disease Control and Prevention
CI: 95% Confidence Interval
CMAQ: Community Multiscale Air Quality Model
ED: Emergency Department
EPA: U.S. Environmental Protection Agency
EPHT: Environmental Public Health Tracking
FRM: Federal Reference Method
GIS: Geographic Information System
HBM: Hierarchical Bayesian Model
HF: Heart Failure
HSCRC: Maryland Health Services Cost Review Commission
ICD-9-CM: International Classification of Diseases, Ninth Revision, Clinical Modification
IP: Inpatient Hospitalization
MDH: Maryland Department of Health
MDP: Maryland Department of Planning
MI: Myocardial Infarction
MODIS: MODerate resolution Imaging Spectroradiometer
LAADS: Level-1 and Atmosphere Archive and Distribution System
NASA: National Aeronautics and Space Administration
NCEI: National Centers for Environmental Information
NOAA: National Oceanic and Atmospheric Administration
OPM: U.S. Office of Personnel Management
OR: Odds Ratio
PHASE: Public Health Air Surveillance Evaluation
PHREG: Proportional Hazards Regression
PM_2.5_: Fine Particulate Matter
PMB: PM_2.5_ Baseline Model (monitor PM_2.5_ and CMAQ PM_2.5_)
PMC: AOD PM_2.5_ Model (monitor PM_2.5_ and AOD PM_2.5_)
PMCK: AOD PM_2.5_ Kriged Model (monitor PM_2.5_ and AOD PM_2.5_ Kriged)
PMCKQ: AOD PM_2.5_ Kriged and CMAQ PM_2.5_ Model (monitor PM_2.5_ and AOD PM_2.5_ Kriged and CMAQ PM_2.5_)
PMCQ: AOD PM_2.5_ and CMAQ PM_2.5_ Model (monitor PM_2.5_ and AOD PM_2.5_ and CMAQ PM_2.5_)
SAS: Statistical Analysis System
USCB: U.S. Census Bureau/Department of Commerce
ZCTA: ZIP Code Tabulation Area (US Census)
ZIP Code: Zone Improvement Plan (US Postal Service)
