# Supplementary material for: Contribution of Satellite-Derived Aerosol Optical Depth PM2.5 Bayesian Concentration Surfaces to Respiratory-Cardiovascular Chronic Disease Hospitalizations in Baltimore, Maryland
Source: Atmosphere (Basel). Author manuscript; Available in PMC 2021 May 11. (PMC8112581; doi:10.3390/atmos11020209)
Supplement: supplementary [file NIHMS1568248-supplement-supplementary.pdf]

Supplemental Material: Contribution of Satellite-Derived Aerosol Optical Depth $PM_{2.5}$ Bayesian Concentration Surfaces to Respiratory-Cardiovascular Chronic Disease Hospitalizations in Baltimore, Maryland

John T. Braggio, Eric S. Hall, Stephanie A. Weber, and Amy K. Huff

Table of Contents: Supplemental (S) Tables and Figures

1) Table S1: CMAQ 12 km^2 grids with the 17 $PM_{2.5}$ ambient air monitors in the Baltimore study.
2) Table S2: PMB and the four experimental aerosol optical depth (AOD)-$PM_{2.5}$ concentration surfaces in the Baltimore study.
3) Table S3: Percentiles (PCTL), means and 95% confidence intervals (CIs) for PMB and the four experimental aerosol optical depth (AOD)-$PM_{2.5}$ concentration surfaces in the Baltimore study and means in the New York City study.
4) Table S4: Correlations (r) and percent of variance (r^2%) between PMB and the four experimental aerosol optical depth (AOD)-$PM_{2.5}$ concentration surfaces by monitor status (All, Yes, No) in the Baltimore study.
5) Table S5: Lag days 0-4 for PMB and the four experimental aerosol optical depth (AOD)-$PM_{2.5}$ concentration surface means and 95% confidence intervals (CIs) measured in $\mu g/m^3$ in the Baltimore Study: All CMAQ grids.
6) Table S6: Lag days 01, 24 and 04 for PMB and the four experimental aerosol optical depth (AOD)-$PM_{2.5}$ concentration surface means and 95% confidence intervals (CIs) measured in $\mu g/m^3$ in the Baltimore study: All CMAQ grids.
7) Table S7: Lag days 0-4 for PMB and the four experimental aerosol optical depth (AOD)-$PM_{2.5}$ concentration surface means and 95% confidence intervals (CIs) measured in $\mu g/m^3$ in the Baltimore study: CMAQ grids with monitors.

8) Table S8: Lag days 01, 24 and 04 for PMB and the four aerosol optical depth (AOD)-$PM_{2.5}$ concentration surface means and 95% confidence intervals (CIs) measured in $\mu g/m^3$ in the Baltimore study: CMAQ grids with monitors.

9) Table S9: Lag days 0-4 for PMB and the four experimental aerosol optical depth (AOD)-$PM_{2.5}$ concentration surface means and 95% confidence intervals (CIs) measured in $\mu g/m^3$ in the Baltimore study: CMAQ grids without monitors.

10) Table S10: Lag days 01, 24 and 04 for PMB and the four experimental aerosol optical depth (AOD)-$PM_{2.5}$ concentration surface means and 95% confidence intervals (CIs) measured in $\mu g/m^3$ in the Baltimore study: CMAQ grids without monitors.

11) Figure S1: Odds ratios (ORs) and 95% confidence intervals (CIs) for PMB and the four experimental aerosol optical depth (AOD)-$PM_{2.5}$ concentration surfaces during the warm and cold seasons at lag day 0: A) ED asthma (top left panel), B) IP asthma (top right panel), C) IP MI (bottom left panel), and D) IP HF (bottom right panel).

12) Figure S2: Odds ratios (ORs) and 95% confidence intervals (CIs) for PMB and the four experimental aerosol optical depth (AOD)-$PM_{2.5}$ concentration surfaces during the warm and cold seasons at lag day 1: A) ED asthma (top left panel), B) IP asthma (top right panel), C) IP MI (bottom left panel) and D) IP HF (bottom right panel).

13) Figure S3: Odds ratios (ORs) and 95% confidence intervals (CIs) for PMB and the four experimental aerosol optical depth (AOD)-$PM_{2.5}$ concentration surfaces during the warm and cold seasons at lag days 01: A) ED asthma (top left panel), B) IP asthma (top right panel), C) IP MI (bottom left panel) and D) IP HF (bottom right panel).

Table S1: CMAQ 12 km^2 grids with the 17 $PM_{2.5}$ ambient air monitors in the Baltimore study[1, 2].

MON NO	ROW, COL	SITE NO	COUNTY	CITY	REC DUR
1	2, 6	8003	Prince George's	Bowie	3 Years
2	3, 5	0002	Prince George's	Greenbelt	<3 Years
3	3, 7	0014	Anne Arundel	Not City	<3 Years
4	4, 3	3001	Montgomery	Not City	3 Years
5	4, 5	0030	Prince George's	Beltsville	3 Years
6	4, 6	0019	Anne Arundel	Fort Meade	<3 Years
7	5, 6	1003	Anne Arundel	Glen Burnie	3 Years
8	5, 7	2002	Anne Arundel	Riviera Beach	<3 Years
9	6, 6	0035	Baltimore	Baltimore	3 Years
10	6, 6	0040	Baltimore	Baltimore	3 Years
11	6, 6	0049	Baltimore	Baltimore	<3Years
12	6, 7	0008	Baltimore	Baltimore	3 Years
13	7, 5	0007	Baltimore	Baltimore	3 Years
14	7, 6	0006	Baltimore	Baltimore	3 Years
15	7, 7	3001	Baltimore	Essex	3 Years
16	8, 6	1007	Baltimore	Cockeysville	3 Years
17	8, 8	1001	Harford	Edgewood	3 Years

[1]All Federal Reference Method (FRM) monitors recorded ambient $PM_{2.5}$ intermittently, usually every 3rd day, by using the gravimetric method.
[2]Column abbreviations: MON NO=Monitor Number; ROW, COL=Row, Column; REC DUR=Recording Duration.

Table S2: $PM_{2.5}$ and the four experimental aerosol optical depth (AOD)-$PM_{2.5}$ surfaces in the Baltimore study.

SURFACE NAME	SURFACE DESCRIPTION	INPUT SURFACES USED[1]
PMB	$PM_{2.5}$ Baseline	Monitor $PM_{2.5}$ & CMAQ $PM_{2.5}$
PMC	AOD $PM_{2.5}$	Monitor $PM_{2.5}$ & AOD $PM_{2.5}$
PMCK	AOD $PM_{2.5}$ Kriged	Monitor $PM_{2.5}$ & AOD $PM_{2.5}$ Kriged
PMCQ	AOD $PM_{2.5}$ & CMAQ	Monitor $PM_{2.5}$ & AOD $PM_{2.5}$ & CMAQ $PM_{2.5}$
PMCKQ	AOD $PM_{2.5}$ Kriged & CMAQ	Monitor $PM_{2.5}$ & AOD $PM_{2.5}$ Kriged & CMAQ $PM_{2.5}$

[1]AOD readings obtained from NASA Aqua and Terra synchronized orbiting satellites and expressed as proportional $PM_{2.5}$ concentration values recorded from on-the-ground $PM_{2.5}$ monitors.

Table S3: Percentiles (PCTL), means and 95% confidence intervals for PMB and the four experimental aerosol optical depth (AOD)-$PM_{2.5}$ concentration surfaces in the Baltimore study and means in the New York City study.

SURF[1-3]	1ST PCTL	25TH PCTL	50TH PCTL	75TH PCTL	99TH PCTL	MEAN	CI 95%	NYC MEAN
PMB	8.54	11.89	14.05	16.23	21.40	14.19	14.13-14.26	10.02
PMC	9.08	11.25	13.05	15.87	20.61	13.66	13.60-13.72	12.03
PMCK	9.85	11.81	13.28	16.89	21.50	14.38	14.31-14.44	10.51
PMCQ	8.70	11.53	13.64	15.72	20.43	13.79	13.73-13.85	10.09
PMCKQ	9.15	11.71	13.64	15.95	20.59	13.91	13.85-13.97	12.91

[1]Column heading abbreviation: SURF=Surface; PCTL=Percentile; CI=95% Confidence Interval; NYC=New York City.
[2]N=8316 grid cells for each surface in Baltimore study.
[3]Three-year mean $PM_{2.5}$ concentration readings in $\mu g/m^3$.

Table S4: Correlations (*r*) and percent of variance (r^2%) between PMB and the four experimental aerosol optical depth (AOD)-$PM_{2.5}$ concentration surfaces by monitor status (All, Yes, No) in the Baltimore study.

SURF[1-3]	ALL r	ALL r^2 %	YES r	YES r^2 %	NO r	NO r^2 %	NO r^2 %- YES r^2 %
PMC	0.676	45.70	0.858	73.62	0.642	41.22	-32.40
PMCK	0.553	30.58	0.788	62.09	0.515	26.52	-35.57
PMCQ	0.973	94.67	0.987	97.42	0.971	94.28	-3.13
PMCKQ	0.852	72.59	0.928	86.12	0.838	70.22	-15.89

[1]Column acronym name: SURF=Surface.
[2]Total observations for each monitor grid group: All, 8316; Yes, 1260; No, 7056.
[3]All correlations significant, $p<=0.01$.

Table S5: Lag days 0-4 for PMB and the four experimental aerosol optical depth (AOD)-$PM_{2.5}$ concentration surface means and 95% confidence intervals (CIs) measured in μg/m^3 in the Baltimore study: All CMAQ grids.

SURF[1]	LAG DAY 0	LAG DAY 1	LAG DAY 2	LAG DAY 3	LAG DAY 4
PMB	14.19 (14.13-14.26)	14.19 (14.13-14.26)	14.19 (14.13-14.26)	14.20 (14.13-14.26)	14.20 (14.13-14.26)
PMC	13.66(13.60-13.72)	13.66 (13.60-13.72)	13.66 (13.60-13.72)	13.66 (13.60-13.72)	13.66 (13.60-13.72)
PMCK	14.38(14.31-14.44)	14.38 (14.31-14.44)	14.38 (14.31-14.44)	14.38 (14.31-14.44)	14.38 (14.31-14.45)
PMCQ	13.79(13.73-13.85)	13.79 (13.73-13.85)	13.79 (13.74-13.85)	13.80 (13.74-13.85)	13.80 (13.74-13.85)
PMCKQ	13.91(13.85-13.97)	13.91 (13.85-13.97)	13.91 (13.85-13.97)	13.91 (13.85-13.97)	13.91 (13.85-13.97)

[1]Column acronym name: SURF=Surface.

Table S6: Lag days 01, 24 and 04 for PMB and the four experimental aerosol Optical depth (AOD)-$PM_{2.5}$ concentration surface means and 95% confidence intervals (CIs) measured in $\mu g/m^3$ in the Baltimore study: All CMAQ grids.

SURF[1]	LAG DAY 01	LAG DAY 24	LAG DAY 04
PMB	14.19 (14.14-14.25)	14.19 (14.14-14.25)	14.19 (14.14.14.24)
PMC	13.66 (13.60-13.72)	13.66 (13.61-13.71)	13.66 (13.60-13.71)
PMCK	14.38 (14.31-14.44)	14.38 (14.32-14.44)	14.38 (14.31-14.43)
PMCQ	13.79 (13.74-13.85)	13.79 (13.74-13.85)	13.79 (13.75-13.84)
PMCKQ	13.91 (13.86-13.97)	13.91 (13.86-13.96)	13.91 (13.87-13.96)

[1]Column acronym name: SURF=Surface.

Table S7: Lag days 0-4 for PMB and the four experimental aerosol optical depth (AOD)-$PM_{2.5}$ concentration surface means and 95% confidence intervals (CIs) measured in μg/m^3 in the Baltimore study: CMAQ grids with monitors.

SURF[1]	LAG DAY 0	LAG DAY 1	LAG DAY 2	LAG DAY 3	LAG DAY 4
PMB	14.60 (14.44-14.76)	14.61 (14.45-14.76)	14.61 (14.45-14.77)	14.62 (14.46-14.78)	14.64 (14.48-14.79)
PMC	13.90 (13.73-14.06)	13.89 (13.73-14.06)	13.89 (13.73-14.06)	13.89 (13.73-14.05)	13.89 (13.73-14.05)
PMCK	14.27 (14.10-14.44)	14.27 (14.10-14.44)	14.27 (14.10-14.44)	14.27 (14.10-14.44)	14.27 (14.10-14.44)
PMCQ	14.28 (14.12-14.43)	14.28 (14.12-14.44)	14.28 (14.13-14.44)	14.29 (14.13-14.44)	14.30 (14.14-14.45)
PMCKQ	14.24 (14.09-14.40)	14.25 (14.09-14.40)	14.25 (14.09-14.40)	14.25 (14.09-14.41)	14.26 (14.10-14.41)

[1]Column acronym name: SURF=Surface.

Table S8: Lag days 01, 24 and 04 for PMB and the four experimental aerosol Optical depth (AOD)-$PM_{2.5}$ concentration surface means and 95% confidence Intervals (CIs) measured in $\mu g/m^3$ in the Baltimore study: CMAQ grids with monitors.

SURF[1]	LAG DAY 01	LAG DAY 24	LAG DAY 04
PMB	14.60 (14.46-14.74)	14.62 (14.49-14.75)	14.61 (14.50-14.73)
PMC	13.89 (13.74-14.05)	13.89 (13.75-14.03)	13.89 (13.76-14.02)
PMCK	14.27 (14.11-14.43)	14.27 (14.12-14.42)	14.27 (14.13-14.41)
PMCQ	14.28 (14.14-14.42)	14.29 (14.16-14.42)	14.29 (14.17-14.40)
PMCKQ	14.24 (14.10-14.39)	14.25 (14.12-14.39)	14.25 (14.13-14.37)

[1]Column acronym name: SURF=Surface.

Table S9: Lag days 0-4 for PMB and the four experimental aerosol optical depth (AOD) $PM_{2.5}$ concentration surface means and 95% confidence Intervals (CIs) measured in $\mu g/m^3$ in the Baltimore study: CMAQ grids without monitors.

SURF[1]	LAG DAY 0	LAG DAY 1	LAG DAY 2	LAG DAY 3	LAG DAY 4
PMB	14.12 (14.05-14.19)	14.12 (14.05-14.19)	14.12 (14.05-14.19)	14.12 (14.05-14.19)	14.12 (14.05-14.18)
PMC	13.62 (13.55-13.68)	13.62 (13.55-13.68)	13.62 (13.55-13.68)	13.62 (13.55-13.68)	13.62 (13.55-13.68)
PMCK	14.39 (14.32-14.47)	14.39 (14.32-14.47)	14.40 (14.32-14.47)	14.40 (14.32-14.47)	14.40 (14.32-14.47)
PMCQ	13.71 (13.64-13.77)	13.71 (13.64-13.77)	13.71 (13.64-13.77)	13.71 (13.64-13.77)	13.70 (13.64-13.77)
PMCKQ	13.85 (13.79-13.92)	13.85 (13.79-13.92)	13.85 (13.79-13.92)	13.85 (13.79-13.92)	13.85 (13.79-13.92)

[1]Column acronym name: SURF=Surface.

Table S10: Lag days 01, 24 and 04 for PMB and the four experimental aerosol optical depth (AOD)-$PM_{2.5}$ concentration surface means and 95% confidence intervals (CIs) measured in $\mu g/m^3$ in the Baltimore study: CMAQ grids without monitors.

SURF[1]	LAG DAY 01	LAG DAY 24	LAG DAY 04
PMB	14.12 (14.06-14.18)	14.12 (14.06-14.18)	14.12 (14.07-14.17)
PMC	13.62 (13.56-13.68)	13.62 (13.56-13.68)	13.62 (13.57-13.67)
PMCK	14.39 (14.32-14.46)	14.40 (14.33-14.46)	14.39 (14.33-14.46)
PMCQ	13.71 (13.65-13.77)	13.71 (13.65-13.76)	13.71 (13.66-13.76)
PMCKQ	13.85 (13.79-13.91)	13.85 (13.80-13.91)	13.85 (13.80-13.90)

[1]Column acronym name: SURF=Surface.

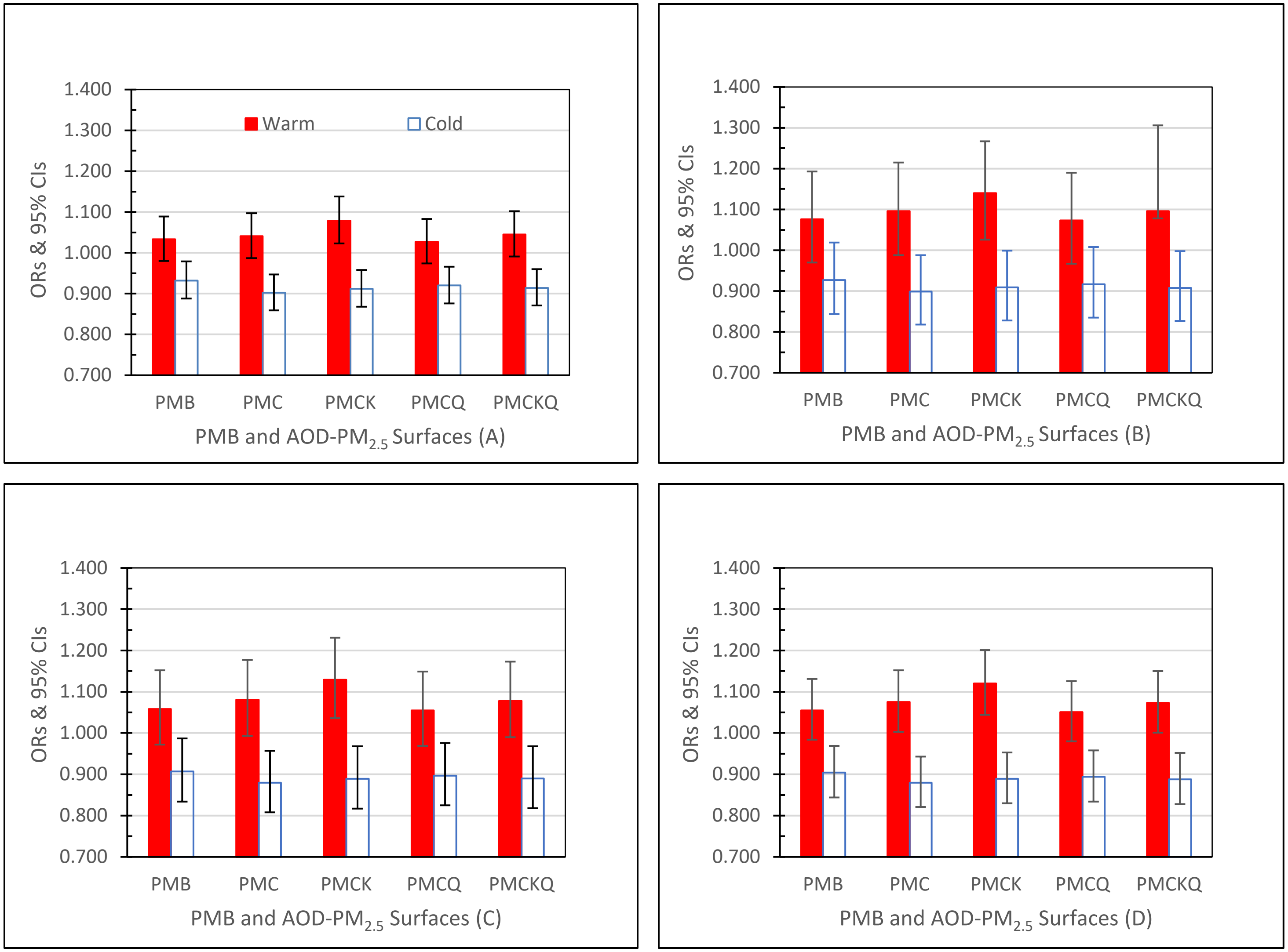

Figure S1: Odds ratios (ORs) and 95% confidence intervals (CIs) for PMB and the four experimental aerosol optical depth (AOD)-$PM_{2.5}$ concentration surfaces during the warm and cold seasons at lag day 0: A) ED asthma (top left panel), B) IP asthma (top right panel), C) IP MI (bottom left panel), and D) IP HF (bottom right panel).

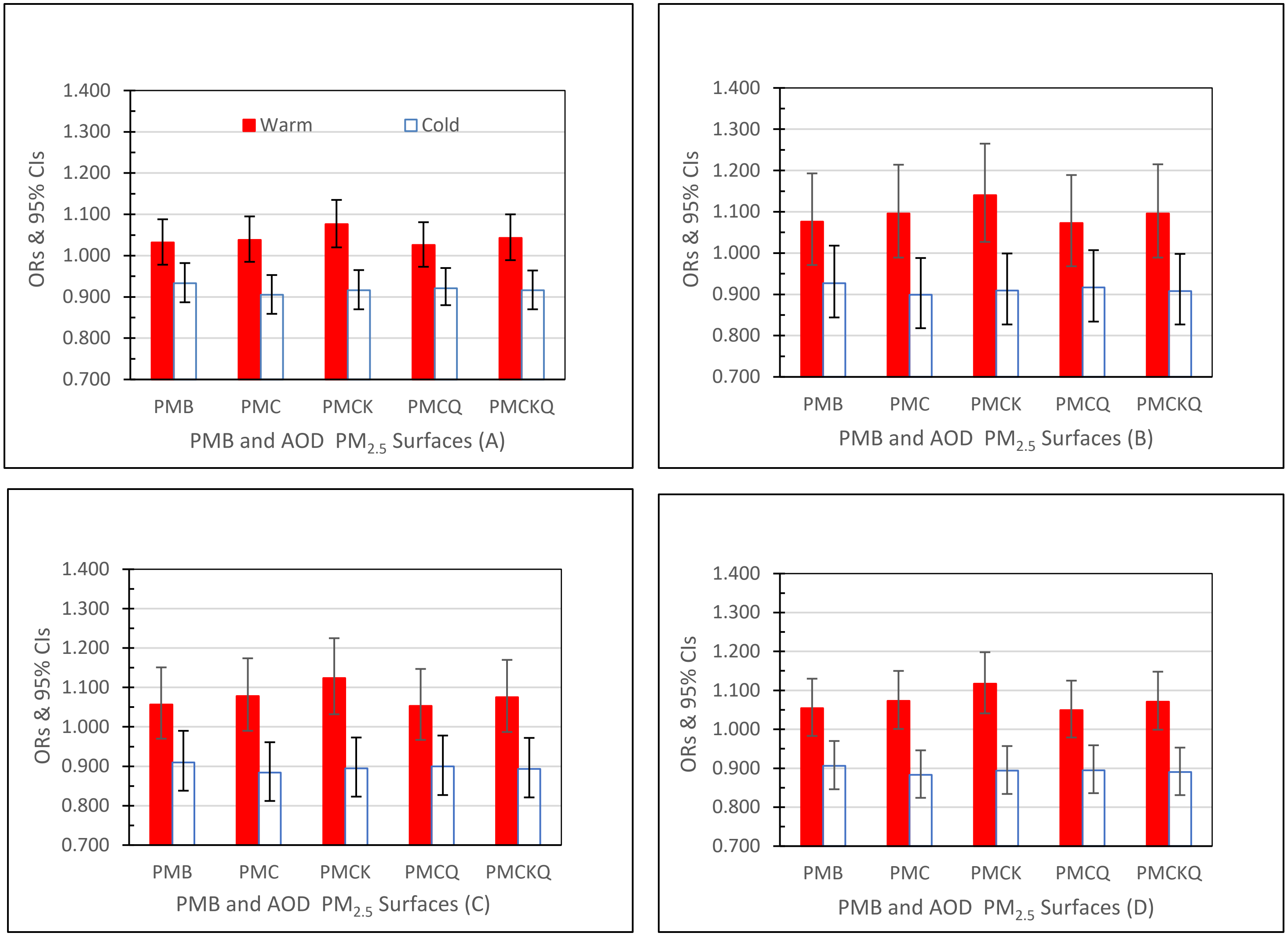

Figure S2: Odds ratios (ORs) and 95% confidence intervals (CIs) for PMB and the four experimental aerosol optical depth (AOD)-$PM_{2.5}$ concentration surfaces during the warm and cold seasons at lag day 1: A) ED asthma (top left panel), B) IP asthma (top right panel), C) IP MI (bottom left panel) and D) IP HF (bottom right panel).

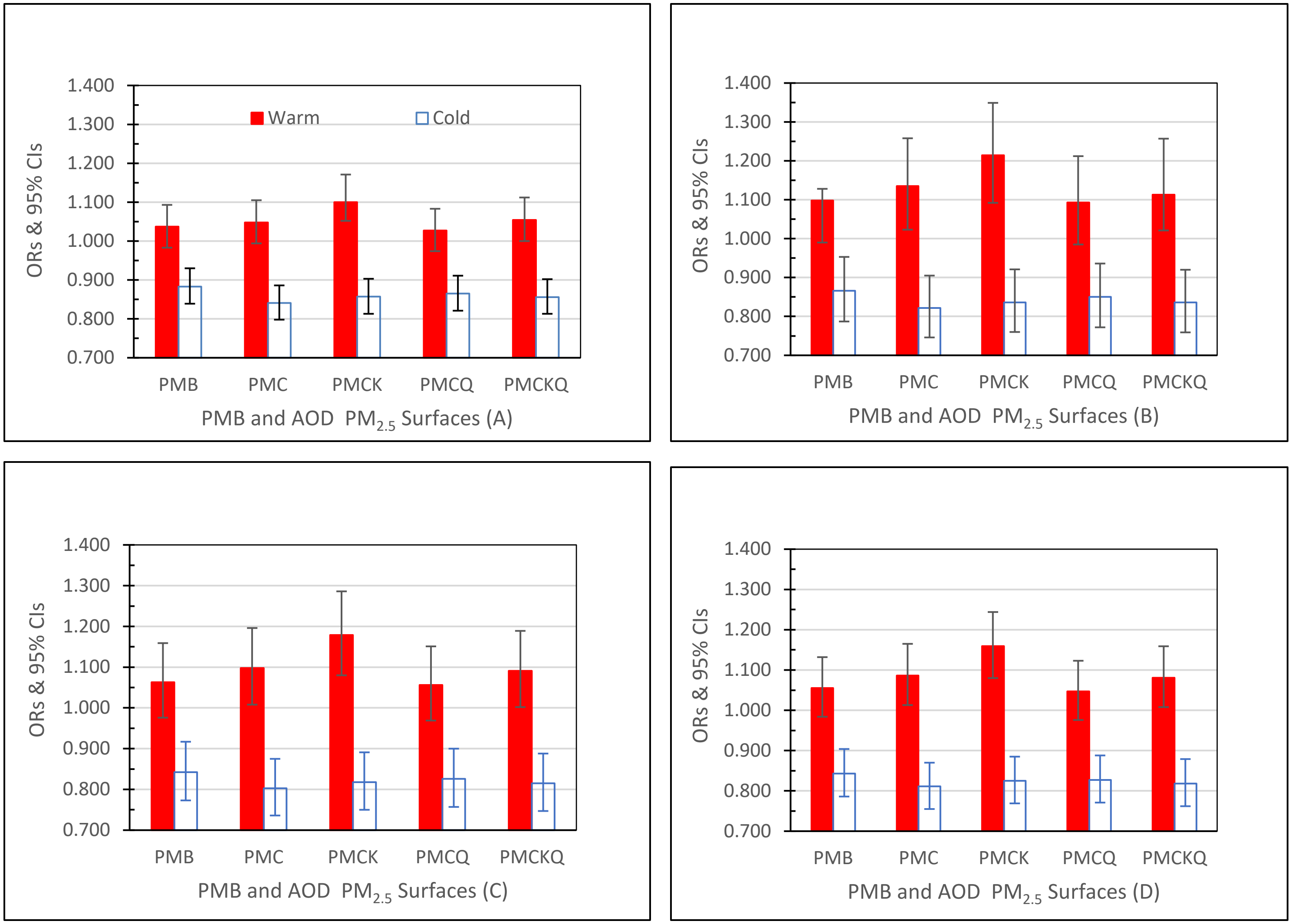

Figure S3: Odds ratios (ORs) and 95% confidence intervals (CIs) for PMB and the four experimental aerosol optical depth (AOD)-$PM_{2.5}$ concentration surfaces during the warm and cold seasons at lag days 01: A) ED asthma (top left panel), B) IP asthma (top right panel), C) IP MI (bottom left panel) and D) IP HF (bottom right panel).
